# Accumulation and penetration behavior of hypericin in glioma tumor spheroids studied by fluorescence microscopy and confocal fluorescence lifetime imaging microscopy

**DOI:** 10.1007/s00216-022-04107-2

**Published:** 2022-05-10

**Authors:** Miriam C. Bassler, Tim Rammler, Frank Wackenhut, Sven zur Oven-Krockhaus, Ivona Secic, Rainer Ritz, Alfred J. Meixner, Marc Brecht

**Affiliations:** 1 Process Analysis and Technology (PA&T), Reutlingen University, Alteburgstr. 150, 72762 Reutlingen, Germany; 2 Institute of Physical and Theoretical Chemistry, University of Tübingen, Auf der Morgenstelle 18, 72076 Tübingen, Germany; 3 Department of Neurosurgery, Schwarzwald-Baar Clinic, Klinikstr. 11, 78052 Villingen-Schwenningen, Germany

**Keywords:** Hypericin, Fluorescence microscopy, Fluorescence lifetime, Photodynamic therapy, Tumor spheroid

## Abstract

Glioblastoma WHO IV belongs to a group of brain tumors that are still incurable. A promising treatment approach applies photodynamic therapy (PDT) with hypericin as a photosensitizer. To generate a comprehensive understanding of the photosensitizer-tumor interactions, the first part of our study is focused on investigating the distribution and penetration behavior of hypericin in glioma cell spheroids by fluorescence microscopy. In the second part, fluorescence lifetime imaging microscopy (FLIM) was used to correlate fluorescence lifetime (FLT) changes of hypericin to environmental effects inside the spheroids. In this context, 3D tumor spheroids are an excellent model system since they consider 3D cell–cell interactions and the extracellular matrix is similar to tumors in vivo. Our analytical approach considers hypericin as probe molecule for FLIM and as photosensitizer for PDT at the same time, making it possible to directly draw conclusions of the state and location of the drug in a biological system. The knowledge of both state and location of hypericin makes a fundamental understanding of the impact of hypericin PDT in brain tumors possible. Following different incubation conditions, the hypericin distribution in peripheral and central cryosections of the spheroids were analyzed. Both fluorescence microscopy and FLIM revealed a hypericin gradient towards the spheroid core for short incubation periods or small concentrations. On the other hand, a homogeneous hypericin distribution is observed for long incubation times and high concentrations. Especially, the observed FLT change is crucial for the PDT efficiency, since the triplet yield, and hence the O_2_ activation, is directly proportional to the FLT. Based on the FLT increase inside spheroids, an incubation time > 30 min is required to achieve most suitable conditions for an effective PDT.

**Graphical abstract:**

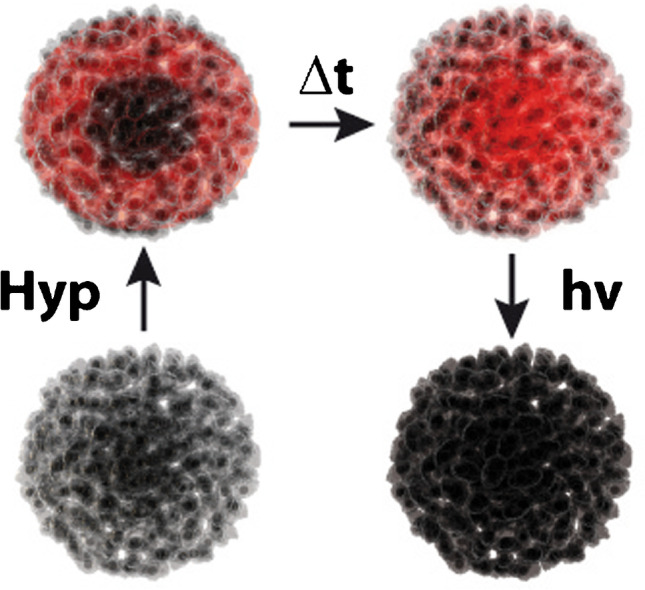

**Supplementary Information:**

The online version contains supplementary material available at 10.1007/s00216-022-04107-2.

## Introduction

Cancer is still one of the most threatening diseases of humankind. In 2020, 19.3 million new cancer cases were diagnosed worldwide [1]. Promising therapeutic and treatment approaches are constantly being developed to prolong the patient’s life and hopefully allow a complete cure. One of these auspicious treatments is photodynamic therapy (PDT) using various photosensitizers, including hypericin [2] that was already characterized chemically [3] and optically [4–7]. In PDT, photosensitizers that enter the excited triplet state upon irradiation either damage the biological substrate directly or generate reactive oxygen species inducing photodamage [8]. So far, PDT with hypericin has been applied to several cancer types, including dermal, colon, and bladder cancer [9, 10]. However, its applicability in glioma tumor treatment necessitates further research.

**Fig. 1:**
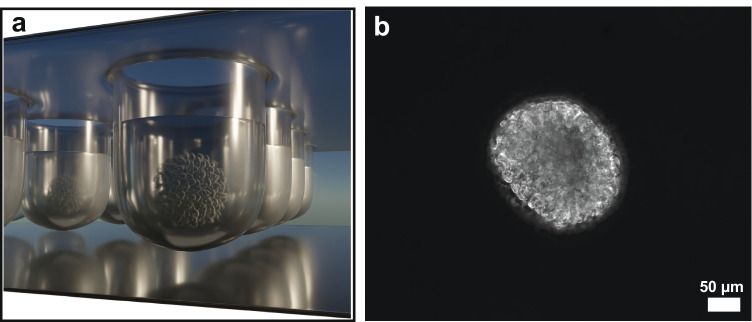
**a** Schematic illustration of the tumor spheroid formation in a 96-well plate by the forced-floating method. Due to a cell-repellent coating inside the well, the included cell suspension is forced to agglomerate to a spheroid. Additionally, the round bottom of the cavities promotes and accelerates the formation of spherical tumor clusters in reproducible size and shape. **b** Phase contrast image of a U-87 MG tumor spheroid with a seeding density of 1500 cells/cm^2^ and a cultivation time of 35 h + 15 min

Most hypericin PDT applications are often tested in 2D cell cultures in vitro [11] after hypericin treatment of the cells. Various studies in cell monolayers showed that hypericin is mainly enriched in the endoplasmic reticulum or Golgi apparatus [12, 13], and others also observed hypericin accumulation in mitochondria or lysosomes [14]. This localization was proven to be dependent on incubation time of hypericin [14]. Cellular uptake mechanisms of hypericin in monolayer experiments were discussed to be either an energy-dependent uptake, e.g., by receptor-mediated endocytosis/pinocytosis [15], or passively driven by diffusion through the cell membrane [16]. Compared to 2D cell cultures, tumor spheroids are a more representative in vitro model, since they better reflect 3D cell–cell interactions and the extracellular matrix (ECM) of tumors in vivo (Fig. 1) [17]. Due to a decreasing nutrient and oxygen supply towards the spheroid center, tumor spheroids can be differentiated into three zones: a proliferative zone at the spheroid’s margin, a transitional zone of quiescent cells, and a necrotic core [18]. Differences between these zones can be ascribed to the cell cycle, as proliferative cells participate in the cell cycle and quiescent cells have left it. Hence, the response of a drug can directly be linked to the cell cycle state of the tumor cells within each zone [19]. Often, a higher response of anticancer drugs was detected in proliferative compared to quiescent cells that are less targeted by the drugs [20]. The drug response can further depend on microenvironmental conditions, like hypoxia, acidosis, complex tumor cell–cell interactions, or the ECM constituents [21], which can be perfectly studied with tumor spheroid models.

The analysis of drug-spheroid interactions is useful for a successful therapeutic implementation, as shown in several studies [22], but only a small number of studies were performed on spheroids and hypericin [23–26]. In this context, the distribution and localization of the drug as well as the cellular uptake and transfer by the tumor spheroids are important [27]. Huygens et al. investigated hypericin penetration in bladder spheroids related to the expression of E-cadherin, a cell molecule contributing to cell–cell adhesion [25]. An additional study involving bladder spheroids concluded that the ECM might also contribute to the hypericin penetration and in this case limit the hypericin intake [24]. Hypericin’s lipophilicity and binding ability to surrounding lipoproteins additionally influence its accumulation and permeation behavior [28]. A deep understanding of these intake and penetration mechanisms in spheroids is essential to realize its full potential for PDT.

An additional impact on PDT performance is expected to result from different local cellular environments throughout tumors and tumor spheroids, caused by the changing availability of nutrients, oxygen, and glucose [22, 29]. Based on the prevalent conditions, like hypoxia or starvation, within spheroids, cells can react differently in terms of metabolism, gene, and protein expression [30, 31], which might directly influence not only the hypericin accumulation but also the effectiveness of PDT. A powerful tool to detect these cellular, environmental variations is fluorescence lifetime imaging microscopy (FLIM). In FLIM, the excited state decay rate of a probe molecule, represented by the fluorescence lifetime (FLT), is measured with the spatial resolution of a confocal microscope and can be related to the local cellular environment [32]. Several studies used FLIM to measure FLT changes of NAD(P)H in 2D and 3D cell culture systems [33, 34] or to reveal the cell cycle S-phase of spheroids [35]. Recent single molecule studies of hypericin show that the local environment has a strong influence on its FLT [4, 6]. However, applications of FLIM with hypericin in general and especially in a cellular context are rare. Nevertheless, FLIM was used to analyze the hypericin release from nanocarriers or pharmaceutical preparations into the intracellular environment [36] and the interaction and localization of hypericin in cells [37, 38]. For this reason, FLIM with hypericin can be used to investigate microenvironmental effects within tumor spheroids, particularly regarding the application of PDT. The obtained knowledge about the cellular environment is essential for the adaptation and optimization of the PDT treatment.

In our study, we use fluorescence microscopy and FLIM to investigate hypericin within glioblastoma tumor spheroids. Fluorescence microscopy allows us to study the accumulation and distribution of hypericin to better understand the extent and dynamics of its penetration into glioma spheroids. Additional analyses of spheroids by FLIM enable the detection of cellular environmental effects due to hypericin FLT changes, from which valuable information about prevalent cellular conditions can be deduced. Since hypericin functions not only as probe molecule for FLIM, but is considered as photosensitizer for PDT at the same time, the detailed analysis of hypericin’s localization and its environmental characterization in the glioma spheroids allows us to understand the impact of both parameters on the hypericin PDT for brain tumors. For our investigations, spheroids are treated for variable incubation times and with different incubation concentrations to examine hypericin uptake, permeation, and FLT. For each incubation condition, spheroid cryosections are prepared and outer and inner sections are compared to study local differences. Our results generate a fundamental understanding of photosensitizer interactions with tumor spheroids as complex biological systems. This knowledge delivers vital information to improve PDT efficiency in tumors and is applicable to other photosensitizers used for PDT treatment.

## Materials and methods

### Cell culture

Tumor spheroids derive from the glioblastoma cell line U-87 MG (ATCC® HTB-14™). U-87 MG spheroids were generated and cultivated in round-bottom, cell-repellent 96-well plates for 24 h (Greiner bio One) at a density of 1500 cells/cm^2^ using the forced-floating method (Fig. 1a). Eagle’s Minimum Essential Medium (MEM, no glutamine and phenol red, Gibco™) serves as cultivation medium and is supplemented with 10% fetal bovine serum (FBS), 1% L-glutamine, 1% sodium pyruvate, and 1% penicillin–streptomycin (all purchased from Gibco™). Cultivation conditions for the U-87 MG spheroids encompassed 37 °C and a 5% CO_2_ atmosphere.

### Hypericin incubation for different incubation times and concentrations

All preparation steps were performed in the dark. Stock solutions of hypericin (PhytoLab GmbH & Co.KG) in DMSO (Molecular Probes™ D12345) with concentrations of 0.1 mM, 0.25 mM, 0.125 mM, 0.05 mM, and 0.005 mM were prepared and frozen at − 20 °C. Aliquots of this stock were freshly thawed for each experiment. After thawing, a 1:100 dilution of hypericin (1 µM, 2.5 µM, 1.25 µM, 0.5 µM, 0.05 µM) in complete cultivation medium and additional 10% FBS was prepared. This procedure was adopted from [15]. Excess medium was removed from each single well and the hypericin/MEM mixture was added at a final concentration of 1 µM for incubation time experiments. Spheroids were incubated for 5 min, 30 min, 125 min, 10 h + 30 min, and 35 h + 15 min in the hypericin cultivation medium, respectively. Varying hypericin concentrations (2.5 µM, 1.25 µM, 0.5 µM, 0.05 µM) were incubated for a predefined time period of 30 min. Non-hypericin-incubated control spheroids were treated with a 1:100 dilution of DMSO in complete cultivation medium and additional 10% FBS for 30 min. Afterwards, the hypericin and control incubation were disrupted by removing the incubation medium and applying three washing steps with phosphate buffered saline (PBS). Following the washing steps, U-87 MG spheroids were directly preserved by fixation. All experiments were executed in triplicate.

### Spheroid fixation and nucleus staining

All preparation steps were performed in the dark. PBS of the spheroid washing steps was discarded, followed by a formaldehyde fixation (ROTI®Histofix 4%) with an incubation time of 30 min. Afterwards, the fixation solution was removed, and spheroids were washed three times with PBS. For cell nucleus staining, Hoechst 33342 was used as fluorescence dye (NucBlue™ Live ReadyProbes™ Reagent (Hoechst 33342), Invitrogen™). The Hoechst 33342 solution was diluted in PBS according to the NucBlue Reagent protocol. This dilution was added to each U-87 MG spheroid and incubated for 20 min at room temperature. Nucleus staining with Hoechst 33342 was not performed for FLIM. After fixation and Hoechst 33342 incubation, a series of three PBS washing steps was applied. Fixed tumor spheroids were stored in PBS in the dark until microtome sectioning.

### Spheroid sectioning

For preparation of microtome sections, tumor spheroids were embedded in the cryoembedding medium OCT (optimal cutting temperature compound, Sakura Finetek™ Tissue-Tek™ O.C.T. Compound). Each spheroid was transferred into one embedding mold filled up with OCT and quickly cooled down with dry ice to prevent water crystal formation. The frozen OCT blocks were cut with a cryomicrotome (Leica CM3050 S cryostat) and cryosections of 5 µm thickness were produced. Temperatures of the cooling chamber and the sample holder were kept at − 30 °C. Subsequent cutting series of the whole spheroids were manufactured to obtain outer and inner sections of the tumor spheroids. All cutting sections were placed onto a coverslip and stored in the dark for further microscopic imaging experiments.

### Fluorescence and brightfield microscopy

Acquisition of fluorescence images was performed with a Zeiss Axio Observer.Z1 fluorescence microscope equipped with a mercury short-arc lamp (HXP-120) and colored LEDs (Colibri light source, Zeiss). For the fluorescence excitation of Hoechst 33342, a Colibri LED with 385 nm was used. The corresponding filter cube (Zeiss filter set 49) had an excitation wavelength range of 335–383 nm, whereas emission was detected between 420 and 470 nm. LED intensity was set to 70% and the exposure time for one Hoechst image was 250 ms. Hypericin fluorescence was excited with the HXP-120 lamp in a filter wavelength region of 580–604 nm and fluorescence emission was detected between 615 and 725 nm (Zeiss filter set 71). The HXP-120 lamp intensity was defined at the highest intensity level and fluorescence images were acquired with 150 ms per image. All fluorescence images were recorded with a 20 × objective lens (Zeiss Plan-Apochromat 20 × /0.80, Ph 2, M27) and fluorescence detection was conducted with an Axiocam 506 microscope camera. Tumor spheroid sections were imaged by z-stacking. Subsequent image processing entailed the application of the extended depth-of-field fusion algorithm using wavelet transform in order to obtain a sharp composite image (Zeiss ZEN 2.3 software, blue edition). Brightfield microscopy was accomplished with a second Zeiss Axio Observer microscope (Z1/7) using a white light LED. All brightfield images were recorded with a 20 × objective lens (Zeiss LD A-Plan 20 × /0.35, Ph 1) and an Axiocam 305 microscope camera. White light LED intensity was set to 11.5% and exposure times per image were equal to 18 ms.

### Fluorescence lifetime imaging microscopy

The time-resolved confocal fluorescence microscope was custom-built. The used light source was a pulsed laser diode (LDH P–C-405, PicoQuant GmbH, Germany) with an excitation wavelength of 405 nm and at a repetition frequency of 40 MHz. The pulse duration was 50 ps. The beam was focused on the sample by an oil immersion objective lens (Zeiss Plan-Apochromat, 100 × , 1.4 Oil DIC, Carl Zeiss AG, Germany). An additional long-pass filter (EdgeBasic™ Long Wave Pass 405) separated the excitation light from the sample emission. Confocal imaging was achieved using a single photon avalanche diode (SPAD; PDM series, Micro Photon Devices, Italy). By coupling the SPAD with a time-correlated single photon counting (TCSPC) unit (HydraHarp 400, PicoQuant GmbH, Germany) and the pulsed laser diode, time-resolved measurements were performed. The scanning stage, SPAD, laser diode, and TCSPC unit were controlled by SymphoTime® software (PicoQuant GmbH, Germany). Time-resolved measurements were also analyzed and evaluated with the SymphoTime® software.

### Fluorescence spectra acquisition

Fluorescence spectra were recorded using a spectrometer (Acton SP300i, Princeton Instruments, USA) with a thermoelectrically cooled CCD camera (PIXIS 100, Princeton Instruments, USA). The wavelength range of the spectrometer was set to 480–760 nm. A long-pass filter (EdgeBasic™ Long Wave Pass 405) was applied to remove the laser signal from the spectra. The acquisition time for each hypericin spectrum was 250 ms. Fluorescence spectra were acquired using Winspec® software (Princeton Instruments, USA).

## Results

**Fig. 2:**
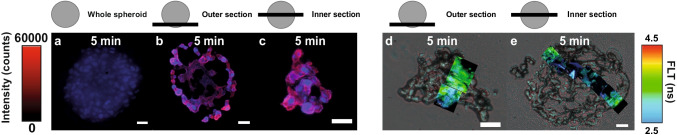
Investigation of hypericin penetration into spheroids by comparing outer and inner spheroid sections. The penetration was examined with fluorescence microscopy and FLIM for an incubation time of 5 min. Fluorescence images of the whole spheroid (**a**) and spheroid sections (**b**, **c**) are displayed. The fluorescence intensity of Hoechst in the nuclei (blue) and hypericin fluorescence (red) is superimposed in **a**–**c**. The outer section reveals a homogeneous hypericin intensity (red) throughout the section compared to the inner one (**b**, **c**). In the inner section, an intensity gradient is observed (**c**). All fluorescence images have scale bars of 25 µm. To represent FLTs across the sections, FLIM images (24 × 24 µm) are superimposed on corresponding brightfield images to generate a composite image (**d**, **e**). Scale bars of the brightfield images represent 25 µm. Hypericin FLTs vary between 3.5 and 2.5 ns depending on the position inside the spheroid (peripheral to central area). An FLT gradient can be observed from the spheroid outside (3.5 ns) to the inside (2.5 ns) (**e**)

A first characterization of whole tumor spheroids was performed by fluorescence microscopy. Figure 2a shows a fluorescence intensity image of a 5-min-incubated tumor spheroid before sectioning. Hypericin fluorescence is shown in red, whereas the nucleus staining with Hoechst 33342 is shown in blue. Based on these colors, cell nuclei and hypericin can be identified in the spheroids (Fig. 2a). Afterwards, cryosections were prepared to analyze and compare the spatial distribution of hypericin in outer and inner spheroid regions in more detail. Figure 2b and c show fluorescence intensity images of outer and inner sections. As a consequence of spheroid cutting, section elongation, compression, and regions without cells can occur. Thus, sections might appear differently shaped or patchy compared to whole spheroids. The technically limited size of the FLIM images (24 × 24 µm) required us to stitch together successive images to cover the entire width of the spheroid sections with FLIM and superimpose them on the respective brightfield images. Figure 2d and e show the respective composite images.

In Fig. 2b, hypericin appears to be homogeneously distributed across the outer spheroid section after 5 min incubation, as visualized by the homogenous fluorescence intensity (Fig. 2b; red). For the inner section, hypericin was mainly accumulated in a peripheral zone of the section and only a small portion penetrated deeper towards the spheroid center (Fig. 2c; red). All observations are corroborated by the single channel fluorescence images in Fig. S1. Control experiments on spheroids without hypericin incubation only reveal the fluorescence intensity of the cell nuclei across the sections (Fig. S2a, SI, blue).

FLIM composite images show a mostly uniform spatial distribution of hypericin FLTs (3.5 ns) throughout the outer section (Fig. 2d). Contrarily, the inner section revealed an FLT gradient across the section. The spheroid edge exhibits an FLT of 3.5 ns, which decreases to 2.5 ns towards the center of the spheroid. As both the hypericin FLT and its intensity are detected simultaneously, these experiments additionally confirm the results obtained by fluorescence microscopy. Although similar trends of hypericin fluorescence intensity and FLT are observable, reasons for both are different since FLTs are concentration-independent, but strongly influenced by the cellular environment [32, 39, 40]. For this reason, hypericin FLTs are used to deduce microenvironmental effects inside the spheroids. FLTs of non-hypericin-treated control spheroids are below 2.5 ns throughout the sections, resulting from the autofluorescence of the spheroids. This represents the untreated spheroid constitution (Fig. S2b, SI).

**Fig. 3:**
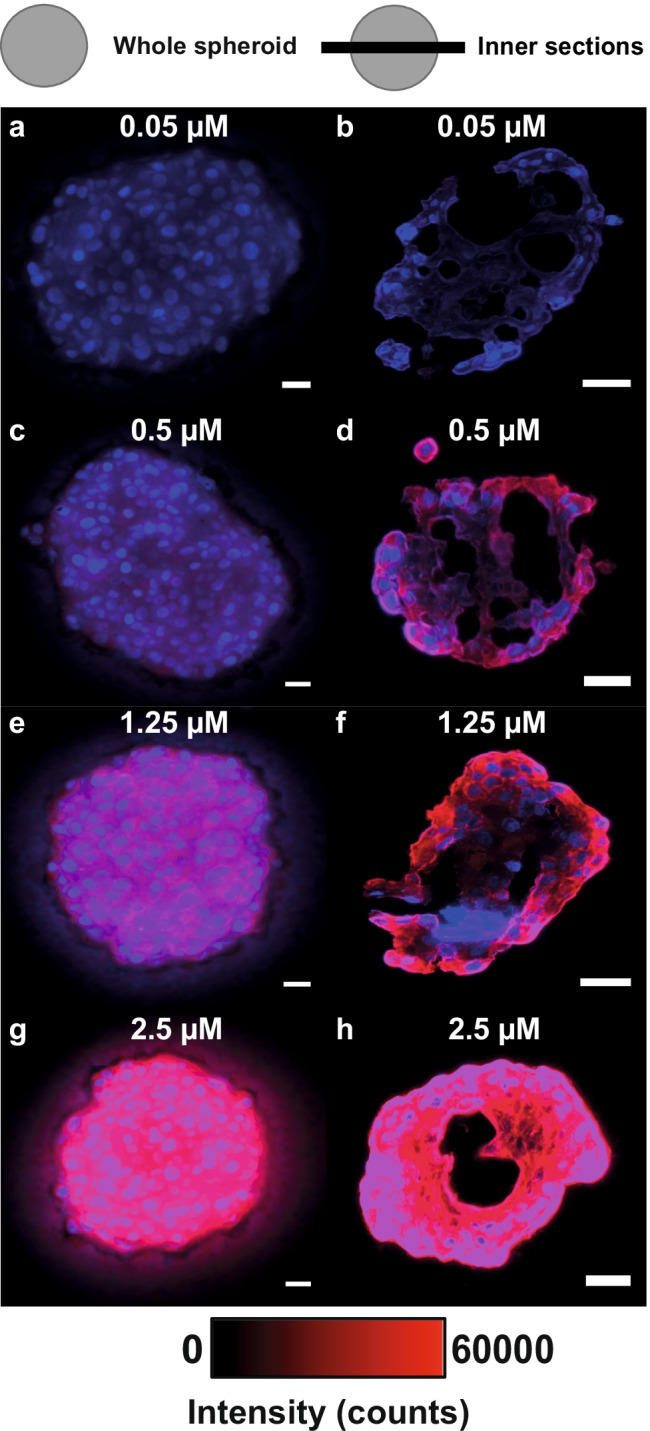
Influence of different incubation concentrations on the hypericin uptake and penetration, investigated by fluorescence microscopy. Fluorescence images of whole spheroids, incubated with different hypericin concentrations (0.05 µM, 0.5 µM, 1.25 µM, 2.5 µM) for 30 min, are illustrated in **a**, **c**, **e**, and **g**. Corresponding fluorescence images of their inner sections are displayed in **b**, **d**, **f**, and **h**. The Hoechst fluorescence intensity in the nuclei (blue) proves cell occurrence throughout the whole spheroids. Hypericin fluorescence intensity (red) of whole spheroids increases with larger incubation concentrations (**a**, **c**, **e**, **g**). As shown by the inner sections, an increase in incubation concentration also results in a higher internal intensity, at least partly, in an annular area at the section edges (**b**, **d**, **f**, **h**; red). Intensity gradients occur for all investigated concentrations (**b**, **d**, **f**, **h**), although hardly visible for the 2.5 µM incubation concentration (**h**). The scale bars of all fluorescence images are 25 µm

This behavior of hypericin in varying spheroid layers points to a difference in uptake and transfer inside the spheroid, which most likely depend on the hypericin incubation concentration. This was successfully shown in fluorescence images of four different hypericin concentrations with a constant incubation time of 30 min, summarized in Fig. 3.

This incubation time was chosen to guarantee a sufficient hypericin amount for fluorescence microscopy, especially at low incubation concentrations. Again, whole spheroids were analyzed prior to cryosectioning (Fig. 3a, c, e, g). As illustrated by the Hoechst staining, cell nuclei are evenly distributed throughout the tumor spheroids (Fig. 3a, c, e, g; blue). Additionally, the fluorescence intensity of hypericin continuously increases with increasing incubation concentration (Fig. 3a, c, e, g; red). Inner sections were prepared to investigate the accumulation and permeation of hypericin inside the spheroids as a function of incubation concentration and are shown in Fig. 3b, d, f, h. Incubation concentrations of 0.05 µM, 0.5 µM, and 1.25 µM show an annular accumulation of hypericin in the outer regions of the spheroid sections (Fig. 3b, d, f; red). Additionally, intensity gradients of hypericin occur towards the spheroid center (Fig. 3b, d, f; red). Although weak, the fluorescence of hypericin at the core is still detectable for these incubation concentrations. The presence of hypericin in central areas was proven by corresponding single channel images in Fig. S3, SI and in spectral line scans in Fig. S4, SI. Incubation with a 2.5 µM hypericin solution caused a high hypericin uptake, indicated by the high fluorescence intensity (Fig. 3h; red). Although hypericin seems to be almost uniformly spread across the whole inner section (Fig. 3h; red), an intensity gradient is also observable, as was confirmed by the single channel images in Fig. S3, SI. Most pronounced fluorescence intensities of hypericin are observed throughout the section for this incubation concentration. Two large holes, caused by the cutting procedure, preclude the innermost hypericin concentration of this section from analysis.

**Fig. 4:**
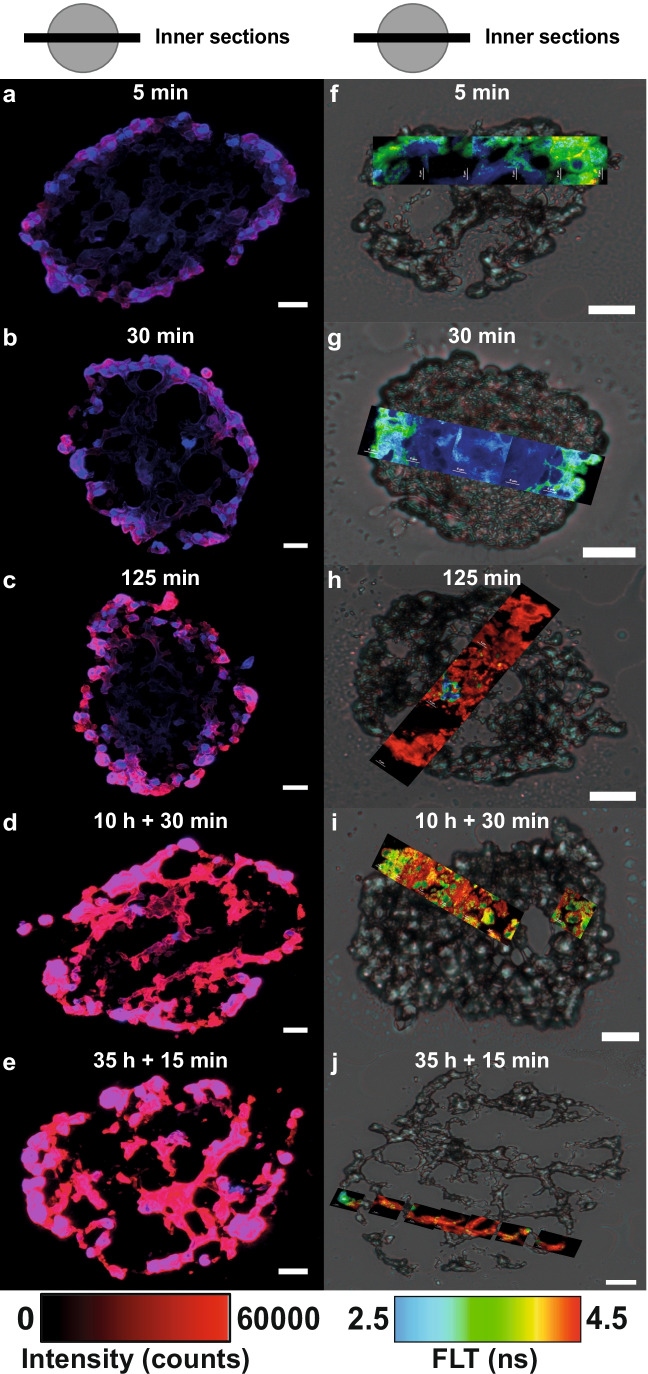
Impact of different incubation times on hypericin at the spheroid center, examined by fluorescence microscopy and FLIM. Inner sections of the spheroids were investigated by both techniques after incubation times of 5 min, 30 min, 125 min, 10 h + 30 min, and 35 h + 15 min, respectively. Cell nuclei were identified by their blue Hoechst fluorescence (**a**–**e**). Fluorescence images of the sections reveal hypericin intensity gradients (red) for a 5 min, 30 min, and 125 min incubation, with decreasing intensity towards the core (**a**–**c**). An evenly distributed hypericin fluorescence intensity is observed for long incubation times of 10 h + 30 min and 35 h + 15 min (**d**, **e**). Additionally, a larger amount of hypericin penetrates deeper into the spheroid at longer incubation times. Overall, an increasing hypericin fluorescence intensity (red) is observed for increasing incubation times (**a**–**e**). The scale bars of all fluorescence images are 25 µm. Composite images were again generated by combining FLIM images (24 × 24 µm) and corresponding brightfield images (**f**–**j**). Corresponding scale bars of the brightfield images also equal 25 µm (**f**–**j**). Composite FLIM images show an FLT range of 2.5–4.5 ns for hypericin throughout the inner sections of the tumor spheroids (**f**–**j**). Gradients of hypericin FLT appear for short incubation times (**f**, **g**), whereas a mostly homogeneous FLT distribution occurs at longer incubation times (**h**–**j**)

Besides the impact of concentration, the duration of hypericin incubation should also influence its uptake, distribution, and FLT inside the spheroids. For this purpose, tumor spheroids were incubated for five different incubation periods (5 min, 30 min, 125 min, 10 h + 30 min, and 35 h + 15 min), using a 1 µM hypericin solution. This concentration was chosen to guarantee a sufficient hypericin amount, especially at short incubation times, for fluorescence microscopy. Fluorescence intensity and FLIM composite images for inner spheroid sections after different hypericin incubation periods are depicted in Fig. 4.

A comparison of inner and outer sections is shown in Fig. S5, SI. FLIM images are again combined with the related brightfield image to create a composite image. Shorter incubation times (5 min, 30 min, 125 min) caused a mainly peripheral hypericin accumulation within the inner section, as can be observed by the higher hypericin fluorescence intensity in this area (Fig. 4a–c). At the center of the tumor spheroid, only a weak hypericin fluorescence intensity is observed, indicating a small hypericin enrichment (Fig. 4a–c). Thus, a similar penetration gradient is noticeable, as previously determined for different incubation concentrations (Fig. 3b, d, f). At even longer incubation times (10 h + 30 min and 35 h + 15 min), the uniform fluorescence intensity shows that hypericin is homogeneously accumulated throughout the whole tumor section (Fig. 4d, e).

FLIM composite images of the 5 min and 30 min incubations revealed an FLT gradient towards the center of the spheroid’s inner sections (Fig. 4f, g). Here, the FLT drops from 3 to 3.5 ns in the outer region to 2.5 ns towards the central area of the sections. However, this gradient is not caused by the hypericin concentration gradient observed in the fluorescence intensity image, but by alterations of the cellular microenvironment. For longer incubation times (125 min, 10 h + 30 min, 35 h + 15 min), the FLTs of hypericin increase to 3–4.5 ns with small areas exhibiting shorter FLTs (2.5–3 ns). Compared to the 5 min and 30 min incubations, no FLT gradient can be observed. Instead, a more homogenous FLT is detected across the spheroid sections (Fig. 4h, i, j). Although similar trends of hypericin fluorescence intensity and FLT are observable, reasons for both are different since FLTs are concentration-independent, but strongly influenced by the cellular environment [32, 39, 40]. For this reason, hypericin FLTs are used to deduce microenvironmental effects inside the spheroids.

**Fig. 5:**
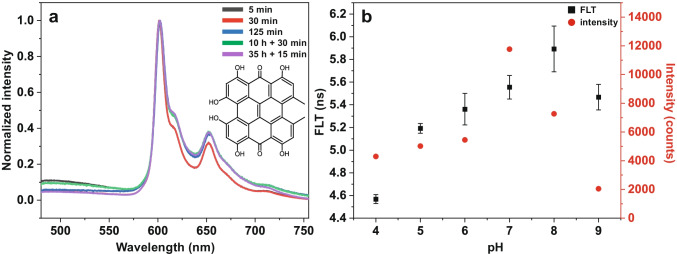
Hypericin fluorescence spectra of inner spheroid sections treated for different incubation times (**a**) and FLTs as well as hypericin maxima intensities depending on pH (**b**). In **a**, fluorescence spectra are averaged line scans across the sections and normalized to maximum intensity. In **b**, FLTs of hypericin and maximum intensities of the 1st hypericin fluorescence maximum (600 nm) are shown for different pH levels (pH 4–9). The largest hypericin FLT of 5.9 ns appears at pH 8, whereas the lowest FLT of 4.5 ns is observed at pH 4. In comparison to FLTs, the highest intensity was measured at pH 7 and the lowest at pH 9

In general, the observed FLT change could occur due to chemical modifications, e.g., deprotonation or isomerization, of hypericin [5, 41, 42]. In order to exclude such effects, fluorescence spectra of hypericin are shown in Fig. 5a as line scans across inner spheroid sections and no alteration of the emission spectra can be observed throughout the sections.

The measurement of line scans additionally confirmed the presence of hypericin all over the sections (Fig. 5a). This was also particularly demonstrated in 2D surface plots for the 5 min incubation in Fig. S4, SI. Hence, a chemical reaction leading to hypericin degradation is unlikely. One factor that potentially impacts hypericin FLTs is the pH, which also shows a gradient towards the spheroid core [43]. Therefore, we investigated FLTs of hypericin in incubation media with different pH levels (pH 4–9). The result is shown by the black squares in Fig. 5b. The physiological pH of the hypericin incubation medium is 8, here the largest hypericin FLT of 5.9 ns can be observed (Fig. 5b, black squares). Decreasing the pH value down to a value of 4 leads to a decrease of the hypericin FLT to 4.5 ns. However, an increase of the pH value to 9 also reduces the hypericin FLT to 5.5 ns (Fig. 5b, black squares). Besides the influence on the FLT, the pH also influences the fluorescence intensity, which is presented as red circles for the emission maximum at 600 nm (Fig. 5b). Corresponding fluorescence spectra at different pH levels are illustrated in Fig. S6, SI. The highest fluorescence intensity at 600 nm is observed at pH 7, whereas the smallest one can be identified for pH 9. Between pH 4 and 6, the fluorescence intensity does hardly change, as reported in literature [44]. Comparable trends of the FLT and the maximum intensity can be observed and highest values for both appear in a physiological pH range of 7–8, decreasing for either lower or higher pH values (pH 4–6, pH 9).

## Discussion

The uptake and the accumulation of hypericin inside U-87 MG glioblastoma cell spheroids using fluorescence microscopy were studied. All results presented by fluorescence microscopy reveal two major phenomena. First, a hypericin gradient is formed from outer to inner spheroid regions for low incubation concentrations or short incubation times, causing an increased hypericin accumulation at the spheroid edge (Figs. 2, 3, 4, S1, and S4, SI). Second, hypericin distributes homogeneously throughout the spheroids for long incubation periods or high concentrations (Figs. 2, 3, 4, S1, and S4, SI). Several effects might be responsible for this accumulation and penetration behavior [23, 26]. Hypericin uptake could be affected by the different spheroid zones, as the drug response can be associated with the cell cycle state that differs in each zone [19]. In the proliferative region, cells pass through the whole cell cycle and are thus more susceptible for drugs, whereas in quiescent areas, cells have left the cell cycle and are consequently less targeted [20]. Thus, the hypericin gradient could be directly related to the cell cycle state. Additionally, metabolic activity also differs between the zones and thus either an active or passive uptake mechanism might be dominant. At the outer spheroid region, an actively driven intake of hypericin due to high cell viability is conceivable, which proceeds to a passive permeation as the metabolic activity decreases in deeper cell layers. These effects can also explain the hypericin fluorescence gradient, as the hypericin concentration continuously decreases between adjacent cell layers due to an inward passive diffusion. With increasing incubation times and concentrations, passive diffusion could cause a larger amount of hypericin to penetrate deeper into the spheroids, as was observed by its homogenous distribution for these incubation conditions. Besides, spheroid-related features, such as cell–cell contacts, ECM [24], and protein activity, e.g., for E-cadherin/P-glycoproteins, might additionally influence hypericin penetration [25]. In any case, we expect passive diffusion of hypericin to be the transfer principle in the necrotic core. Comparable to our results, a similar uptake behavior was also observed for bladder spheroids [23].

In addition to fluorescence microscopy, FLIM was applied to study hypericin FLT differences, which allow to draw conclusions about the cellular environment within spheroids [45]. Compared to the FLT of hypericin on glass (6.34 ± 2.98 ns) or in PVA (4.23 ± 0.89 ns) [4], FLTs are overall shorter in tumor spheroids (2.5–5 ns). This only partially correlates with the refractive indices predominant in cells (nucleus: 1.355–1.365, cytosol: 1.360–1.390, mitochondria: 1.400–1.420) [46]. Since cellular refractive indices are similar to the one of PVA (1.48), comparable hypericin FLTs of approx. 4 ns would be expected [47, 48]. However, most hypericin FLTs in spheroid sections are below 4 ns, so additional factors must be considered. As FLTs are independent of local probe concentration [39], the observed FLT gradients do not result from different hypericin concentrations across the spheroid. Instead, FLTs are strongly dependent on the microenvironment, including the pH level. Based on the spheroid’s structure, a diminished supply with nutrients/oxygen [49] and a hampered removal of metabolic waste [22] cause hypoxia in the core and the lactate production increases [43, 50]. This leads to a lactate-induced acidosis and the pH decreases towards the spheroid center (pH 6–7) [43]. Therefore, the pH inside the spheroid can affect the FLT of hypericin. Figure 5 clearly demonstrates that a decreasing pH is accompanied by a decline in hypericin FLT, which can, at least partly, explain the FLT gradients for short incubation times. However, the FLT decrease is larger and the average FLT is smaller in the spheroids than the ones observed in Fig. 5b, hence additional parameters need to be considered. Associated with hypoxia and acidosis, cellular metabolism changes in deeper spheroid layers, where lactate serves as an alternative substrate for ATP synthesis [51]. The altered metabolism inside the spheroids might also cause a change in microenvironment and thus influence the FLT gradient for short incubation periods. Therefore, hypericin FLTs could be utilized to reveal metabolic activity inside spheroids. However, these assumptions only seem to be true for hypericin-treated spheroids for short incubation times (5 min, 30 min). For long incubation times, a homogeneous FLT distribution with no gradient towards the center can be observed. However, the FLT is decreased in the outer layers compared to inner ones (Fig. 4i, Fig. S3s, t, SI). In part, this is also observed for the 35 h + 15 min incubation in Fig. 4j. In general, FLTs are independent of the probe concentration [39], but this is not true for excessively high concentrations, where most molecules are agglomerated [38]. Non-fluorescent hypericin aggregates are formed that cause fluorescence self-quenching and decrease the FLT at the spheroids edge. The impact of fluorescence quenching, however, might not be sufficient to describe the FLT behavior completely. The occurrence of intermolecular FRET from hypericin monomers to aggregates [38] or hypericin-hypericin excimer-like interactions [52] could additionally contribute to the FLT decrease at the spheroid’s margin. Additionally, the incubation time was shown to alter the hypericin accumulation in cell organelles, causing a shift of the primary accumulation site from mitochondria to lysosomes for U-87 MG monolayers after 6 h (500 nM) [14]. Hypericin-enriched lysosomes (2 h incubation, 2.5 µM) in U-87 MG were also observed in our experiments (data not shown). In spheroids, the outer regions are incubated comparatively longer than inner ones as hypericin permeates towards the core. Thus, hypericin might predominantly accumulate in lysosomes of the outer spheroid layers, whereas it is mostly located in mitochondria in the inner spheroid. Furthermore, the metabolic activity is increased in the outer regions, which might also favor the redistribution of hypericin towards lysosome uptake. This lysosome enrichment can decrease the hypericin FLT due to an increased refractive index of 1.600 compared to 1.410 in mitochondria [46]. These effects, influencing the excited states lifetimes, have a direct impact on the PDT efficiency, since the triplet formation yield is directly proportional to the FLT [53]. Hence, long FLTs promote the transition to the triplet state, resulting in an increased O_2_ activation and higher PDT efficiency. To achieve long FLTs inside glioblastoma spheroids, incubation times of at least 30 min are required to ensure appropriate conditions for PDT treatment.

## Conclusion

In this study, we examined the accumulation and penetration behavior of hypericin in U-87 MG tumor spheroids and correlated FLT changes of hypericin to cellular, environmental conditions. Here, hypericin was simultaneously regarded as probe molecule for FLIM and as photosensitizer for PDT. Hypericin uptake and FLTs were studied in spheroid cryosections, as a function of hypericin concentration and incubation time. Low incubation concentrations and short incubation times caused an increased enrichment at the peripheral region of the spheroids with hypericin gradients towards the center. These FLT gradients were explained by pH or metabolism-associated influences, as the FLT is independent of the actual concentration for small hypericin concentrations. In this context, active or passive internalization mechanisms as well as other spheroid-related attributes are discussed to be the driving force in hypericin uptake and permeation. In contrast, high incubation concentrations and long incubation periods lead to a more homogeneous hypericin distribution throughout the spheroid. However, short FLTs in the peripheral spheroid regions were explained by agglomeration-based fluorescence quenching and possible FRET and excimer-like interactions as a consequence of excessive hypericin accumulation. A further explanation was deduced from the incubation-time-dependent enrichment of hypericin in different cell organelles. The knowledge about hypericin FLTs can be used to significantly influence PDT, since the triplet formation yield, and thus the O_2_ activation, is directly proportional to the FLT. Hence, small FLTs after short incubations are indicative of an insufficient PDT inside tumor spheroids. Therefore, incubation times of at least 30 min are required to achieve an increased hypericin FLT within spheroids and thus improve the efficiency of PDT. Our findings reveal important insights concerning the localization and state of hypericin in an improved in vitro tumor model, which is essential in order to understand the effects of hypericin PDT for glioma tumors.

## Supplementary Information

Below is the link to the electronic supplementary material.

Supplementary file1 (DOCX 3086 KB)

## Data Availability

The data sets generated and/or analyzed during the current study are not publicly available since they are part of an ongoing PhD thesis. However, the data sets are available from the corresponding authors on reasonable request.
