# Supplementary material for: Accumulation and penetration behavior of hypericin in glioma tumor spheroids studied by fluorescence microscopy and confocal fluorescence lifetime imaging microscopy
Source: Anal Bioanal Chem. 2022 May 10;414(17):4849–60. doi: 10.1007/s00216-022-04107-2 (PMC9234035; doi:10.1007/s00216-022-04107-2)
Supplement: Supplementary file 1 — Supplementary file1 (DOCX 3086 KB) [file 216_2022_4107_MOESM1_ESM.docx]

**Accumulation and penetration behavior of hypericin in glioma tumor spheroids studied by fluorescence microscopy and confocal fluorescence lifetime imaging microscopy**

*Miriam C. Bassler^1,2^, Tim Rammler^2^, Frank Wackenhut^1^, Sven zur Oven-Krockhaus^2^, Ivona Secic^1^,*

*Rainer Ritz^3^, Alfred J. Meixner^2^, Marc Brecht^1,2^*

*Corresponding authors e-mails: frank.wackenhut@reutlingen-university.de, marc.brecht@reutlingen-university.de

*^1^Process Analysis and Technology (PA&T) Reutlingen University, Alteburgstr. 150, 72762 Reutlingen, Germany*

*^2^Institute of Physical and Theoretical Chemistry University of Tübingen, Auf der Morgenstelle 18, 72076 Tübingen, Germany*

*^3^Department of Neurosurgery, Schwarzwald-Baar Clinic, 78052 Villingen-Schwenningen, Germany*

**Keywords**

hypericin, fluorescence microscopy, fluorescence lifetime, photodynamic therapy, tumor spheroid

**Supplementary information**

For an improved visualization of the hypericin accumulation in spheroids, fluorescence images of hypericin and Hoechst 33342 are displayed as separated channel images for outer and inner sections of the 5 min incubation period (Fig. S1a‑d). Single channel images are used to proof the presence of hypericin throughout each section, since the hypericin emission is sometimes hardly visible in the fluorescence images in Fig. 2.


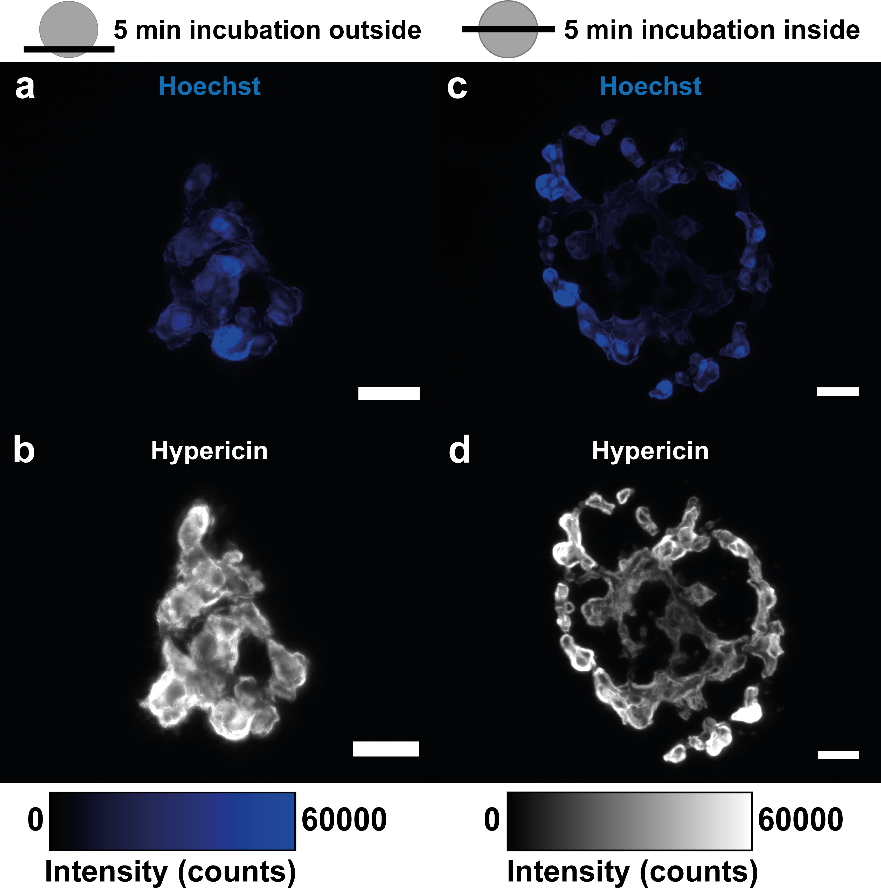


**Figure S1** Verification and illustration of hypericin in outer and inner spheroid sections for a 5 min incubation period. Hoechst 33342 fluorescence by cell nuclei (blue) and hypericin fluorescence (white) are shown separately for outer and inner sections, respectively (a, c and b, d). Single channel fluorescence images of hypericin are used to demonstrate that hypericin is present throughout the sections, indicated by the white color (b, d). For a 5 min incubation, the outer section reveals a more homogeneous hypericin intensity distribution compared to the inner one (b, d), exhibiting a hypericin intensity gradient. Intensity drops in-between result from holes in the sections. As a consequence of section preparation, cutting elongation, compression and regions without cells can occur. Thus, sections might appear differently shaped or patchy. The scale bars of all fluorescence images are 25 µm.

Control spheroids without hypericin treatment were additionally analyzed by fluorescence intensity imaging and FLIM (Fig. S2). For fluorescence intensity imaging, control spheroids were stained with Hoechst 33342 to reveal cell nuclei inside the investigated sections (Fig. S2a). Besides the Hoechst fluorescence, no additional fluorescence was detectable. FLIM analysis showed homogeneously distributed FLTs mostly below 2.5 ns across control sections (Fig. S2b). Both techniques demonstrate large deviations from hypericin-incubated spheroids in terms of fluorescence intensity and FLT.


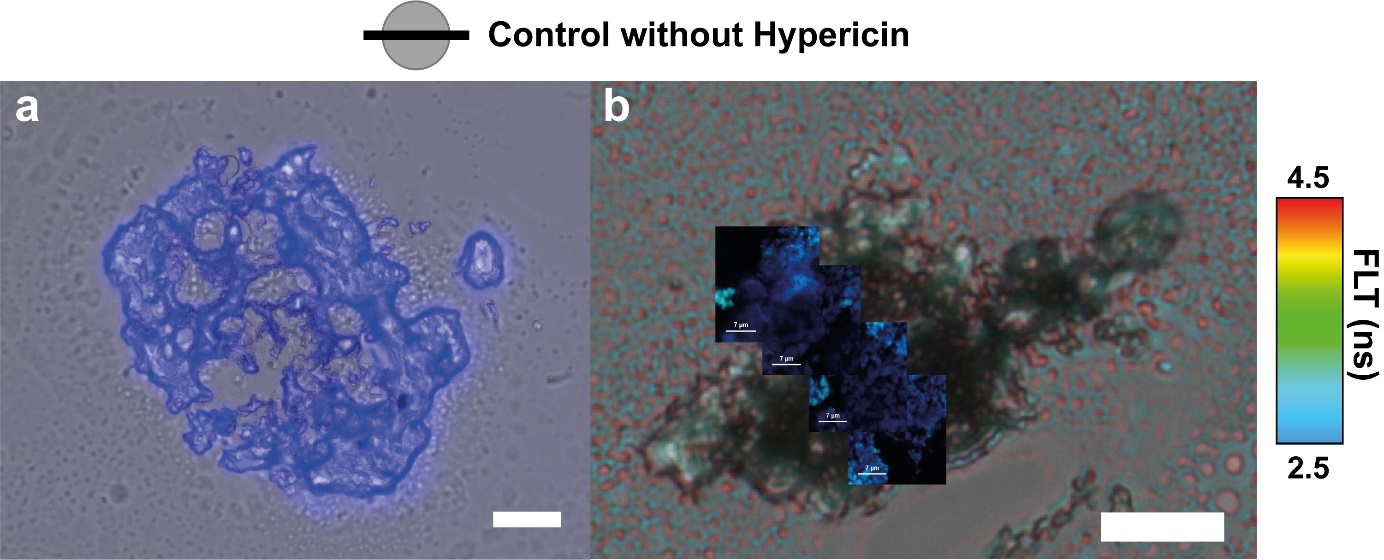


**Figure S2** Control spheroids without hypericin incubation analyzed by nucleus fluorescence intensity and FLIM. In a: Hoechst 33342 nucleus stained fluorescence image (blue) overlaid on top of a brightfield image of the same spheroid section. The scale bar of the fluorescence image is 25 µm. In b: Composite image of a control spheroid with a FLT below 2.5 ns. Composite images were generated by combining FLIM images (24 x 24 µm) and corresponding bright field images. To enable a precise assignment and good visualization of the FLIM images, brightfield images are zoomed-in, thus the sections appear larger. The corresponding scale bar of the brightfield image equals 25 µm. Both techniques demonstrate a clear absence of hypericin inside the spheroid.

Comparable to Fig. S1, separated fluorescence channel images of hypericin (Fig. S3b, d, f, h) and Hoechst 33342 (Fig. S3a, c, e, g) are depicted in Fig. S3 for the investigated incubation concentrations. Again, hypericin single channel images are utilized to examine whether hypericin was enriched at the inner section center.


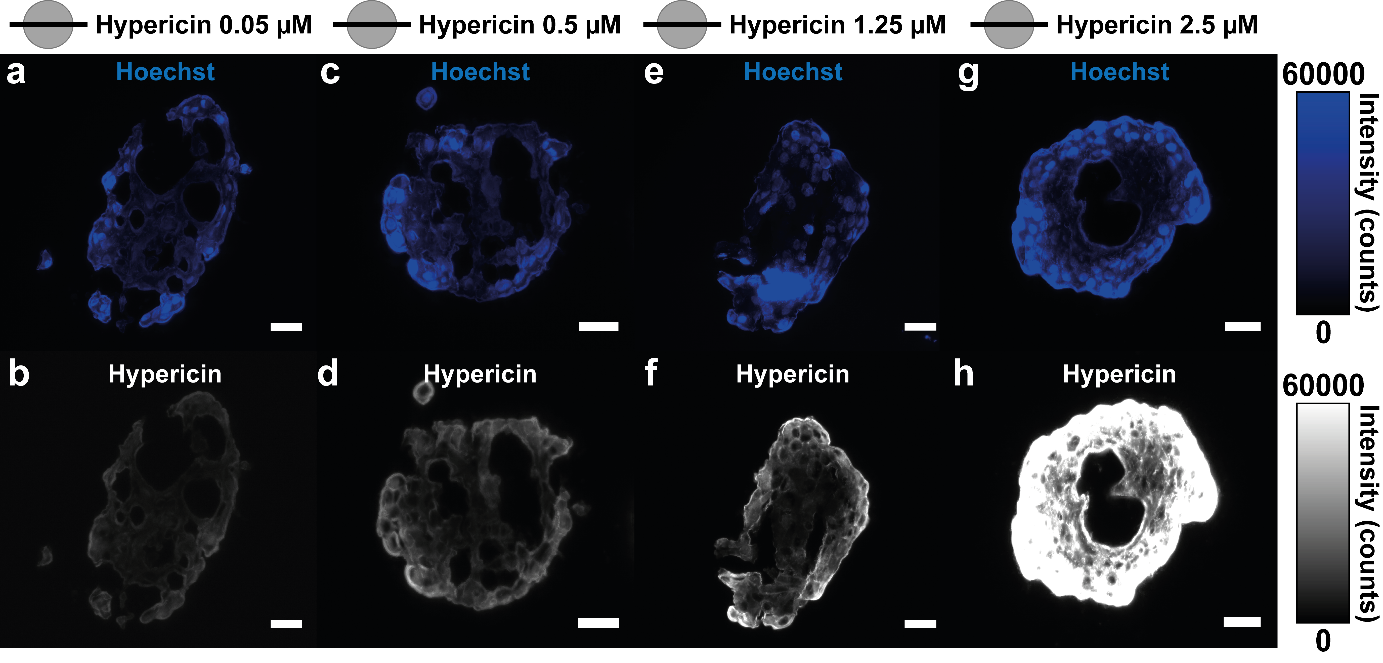


**Figure S3** Verification and illustration of hypericin in inner spheroid sections for different hypericin incubation concentrations. Hoechst 33342 fluorescence by cell nuclei (blue) and hypericin fluorescence (white) are exhibited separately for inner sections (a, c, e, g and b, d, f, h). Single channel fluorescence images of hypericin are used to demonstrate that hypericin also penetrated to the spheroid core, indicated by the white color (b, d, f, h). Inner spheroid sections of all incubation concentrations (0.05 µM, 0.5 µM, 1.25 µM, 2.5 µM) reveal hypericin intensity gradients. Here, 0.05 µM and 0.5 µM concentrations result in a small hypericin intensity increase at the annular area of the sections, but overall hypericin intensities are low (b, d). Within the peripheral regions of the 1.25 µM and 2.5 µM sections, hypericin is accumulated to a larger extent, indicated by the higher fluorescence intensity in these regions (f, h). Inside the 1.25 µM section, hypericin is present in considerably smaller amounts compared to the annular outer areas. For the 2.5 µM section, a continuously decreasing hypericin intensity towards the center is observable. This section reveals highest hypericin intensities compared to all investigated concentrations. As a consequence of section preparation, cutting elongation, compression and regions without cells can occur. Thus, sections might appear differently shaped or patchy. The scale bars of all fluorescence images are 25 µm.

In order to prove the presence of hypericin in the entire inner spheroid sections, fluorescence spectra were acquired across the inner sections. A line scan across the section of a 5 min incubated spheroid is illustrated as 2D surface plot in Fig. S4. This plot displays the fluorescence maxima of hypericin at 600 nm and 650 nm in varying intensities and the y‑axis represents the lateral position in the section. The spectral data demonstrates that hypericin is present all over the sections (Fig. S4a, b).


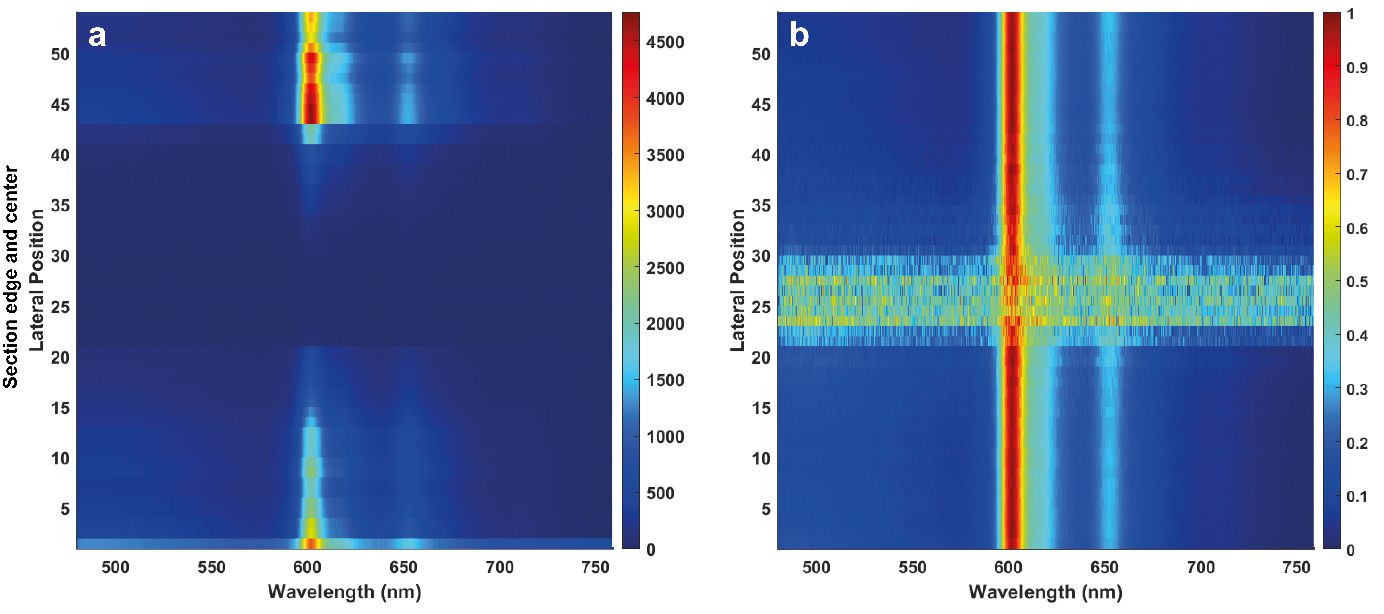


**Figure S4** 2D surface plots of spectral line scans across inner spheroid sections after 5 min incubation with hypericin. In a, the plot is shown with a color scale bar of intensity values in counts, whereas in b, the same spectra are shown with normalized intensity values. In both plots, hypericin fluorescence maxima at 600 nm and 650 nm are visible at all lateral positions throughout the inner section. At the section edges, overall higher hypericin intensities are detectable compared to the section center, thus displaying the intensity gradients observed for short incubation times. In the section center (between lateral position 15 and 40), almost no hypericin intensity is visible in a. However, the normalized intensity plot in b demonstrates that hypericin can be detected throughout the whole section, although its intensity and thus accumulation in the section center is small.

Hypericin in different tumor spheroid regions was investigated by comparing outer and inner sections after varying spheroid incubation times, illustrated in Fig. S5. This was examined by fluorescence intensity imaging and FLIM. Initially, fluorescence intensity images of whole spheroids are shown prior to section preparation (Fig. S5, 1^st^ column). A comparison of fluorescence images between outer and inner sections reveals the reduced accumulation of hypericin towards the spheroid core (Fig. S5, 2^nd^ and 3^rd^ column). On the other hand, FLIM reveals cellular environmental differences from the spheroid outside to the inside due to hypericin FLT changes (Fig. S5, 4^th^ and 5^th^ column). An overview of all outer and inner spheroid comparisons with different incubation durations is summarized in Fig. S5.


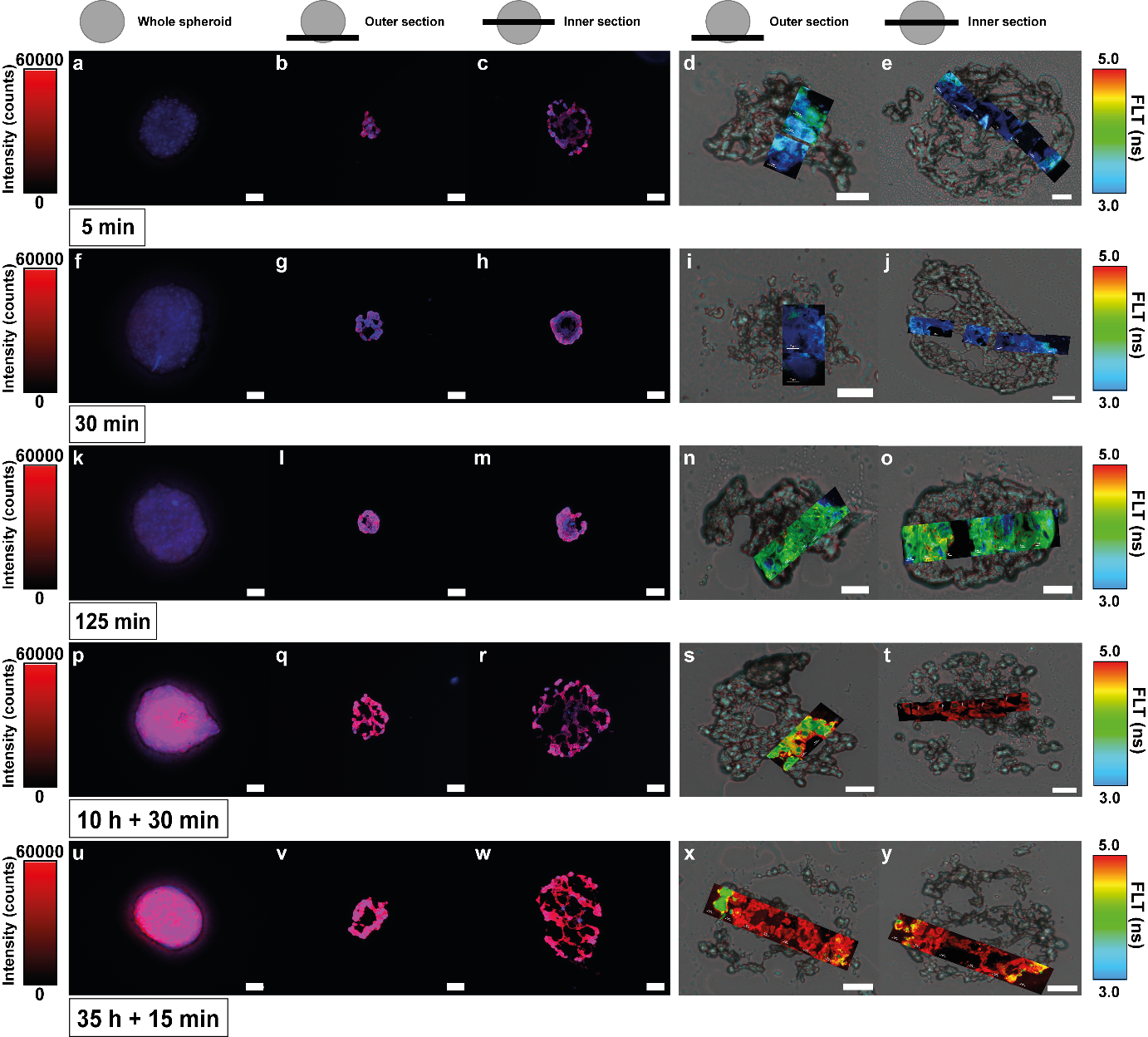


**Figure S5** Comparison of outer and inner spheroid sections after different incubation periods, analyzed by fluorescence intensity imaging and FLIM. Cell nuclei are stained with Hoechst 33342 and illustrated by the blue color, whereas hypericin fluorescence is shown in red color. Fluorescence imaging of whole spheroids already indicates an increasing hypericin enrichment by the more intense red color at longer incubation times (a, f, k, p, u). With increasing incubation duration, hypericin accumulates not only in the outer spheroid layers to an increasing extent, but also towards the spheroid center (b, g, l, q, v and c, h, m, r, w). For short incubation durations (5 min, 30 min, 125 min), hypericin gradients towards the spheroid core are noticeable (c, h, m), whereas longer incubation periods (10 h + 30 min, 35 h + 15 min) result in a more homogeneous hypericin distribution (r, w). The scale bars of all fluorescence images are 50 µm. For FLIM investigation, composite images were generated by combining FLIM images (24 x 24 µm) and corresponding bright field images. To enable a precise assignment and good visualization of the FLIM images, brightfield images are zoomed-in, thus the sections appear larger. Corresponding scale bars of the brightfield images are equal to 25 µm (white). Composite images reveal FLT gradients from outer to inner spheroid sections for short incubation times (5 min, 30 min) (d, i and e, j). The 125 min incubation displays homogeneous FLT distributions for both sections (n, o) with FLTs of 4 ns. Longer incubation durations (10 h + 30 min, 35 h + 15 min), however, reveal an inversion of hypericin FLTs, since outer sections show, at least partially, shorter FLTs of 4 ns (s, x) compared to inner ones (5 ns; t, y). These short FLTs appear either at the section edge or throughout the whole section (s, x). As a consequence of section preparation, cutting elongation, compression and regions without cells can occur. Thus, sections might appear differently shaped or patchy.

Due to the expectation of pH changes towards the spheroid core, fluorescence spectra and FLTs of hypericin are analyzed at different pH levels. Fig. S6 shows the corresponding hypericin fluorescence spectra depending on pH. The fluorescence intensity of hypericin is affected by the pH alterations in the incubation medium with hypericin and the largest fluorescence intensity is observed at pH 7.


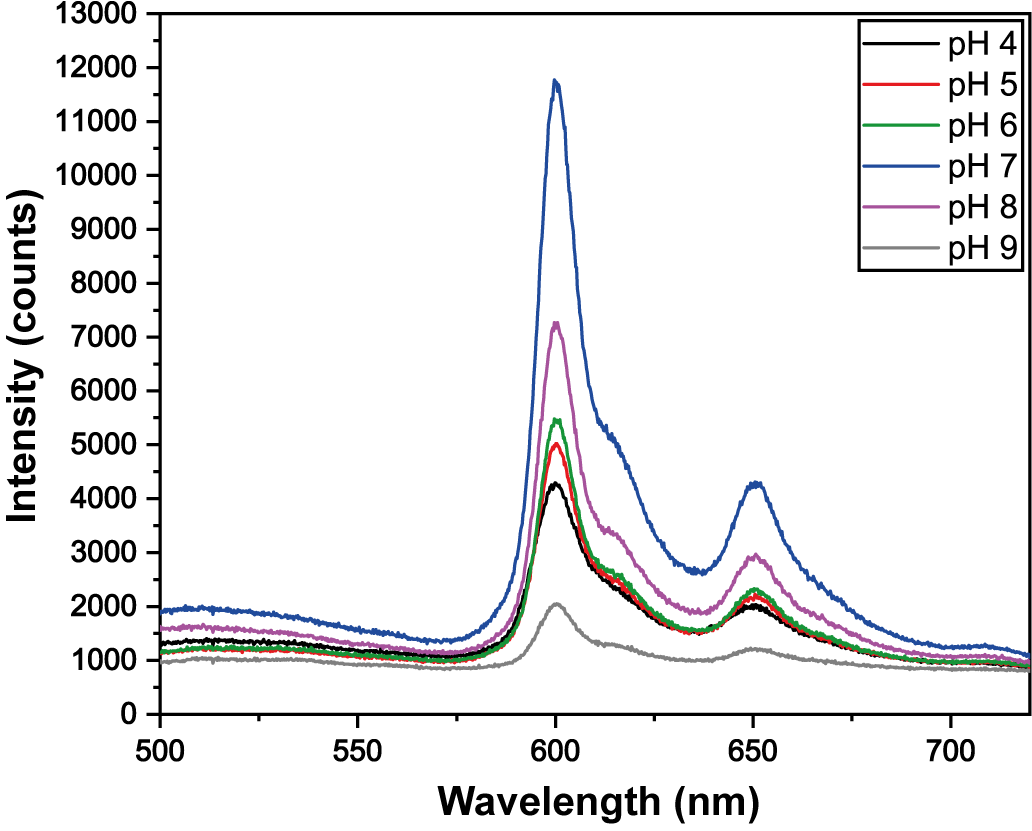


**Figure S6** Hypericin spectra of the used incubation media with pH values adjusted between 4 and 9. Highest hypericin fluorescence intensities are observed for a physiological pH of 7 and 8, whereas the lowest intensity occurs at pH 9.
